# Identifying Ear Abnormality from 2D Photographs Using Convolutional Neural Networks

**DOI:** 10.1038/s41598-019-54779-7

**Published:** 2019-12-03

**Authors:** Rami R. Hallac, Jeon Lee, Mark Pressler, James R. Seaward, Alex A. Kane

**Affiliations:** 1 Department of Plastic Surgery, UT Southwestern, 5323 Harry Hines Blvd., Dallas, TX 75390 United States; 2 Analytical Imaging and Modeling Center, Children’s Medical Center, Dallas, 1935 Medical District Dr., Dallas, Texas 75235 United States; 3 Department of Bioinformatics, UT Southwestern, 5323 Harry Hines Blvd., Dallas, TX 75390 United States

**Keywords:** Diagnosis, Paediatric research

## Abstract

Quantifying ear deformity using linear measurements and mathematical modeling is difficult due to the ear’s complex shape. Machine learning techniques, such as convolutional neural networks (CNNs), are well-suited for this role. CNNs are deep learning methods capable of finding complex patterns from medical images, automatically building solution models capable of machine diagnosis. In this study, we applied CNN to automatically identify ear deformity from 2D photographs. Institutional review board (IRB) approval was obtained for this retrospective study to train and test the CNNs. Photographs of patients with and without ear deformity were obtained as standard of care in our photography studio. Profile photographs were obtained for one or both ears. A total of 671 profile pictures were used in this study including: 457 photographs of patients with ear deformity and 214 photographs of patients with normal ears. Photographs were cropped to the ear boundary and randomly divided into training (60%), validation (20%), and testing (20%) datasets. We modified the softmax classifier in the last layer in GoogLeNet, a deep CNN, to generate an ear deformity detection model in Matlab. All images were deemed of high quality and usable for training and testing. It took about 2 hours to train the system and the training accuracy reached almost 100%. The test accuracy was about 94.1%. We demonstrate that deep learning has a great potential in identifying ear deformity. These machine learning techniques hold the promise in being used in the future to evaluate treatment outcomes.

## Introduction

Congenital auricular deformities occur in 5% of the pediatric population. Patients with ear deformity may undergo neonatal ear molding or surgical correction to improve ear aesthetics and quality of life^1,2^. Plastic surgeons often rely on 2D photography to document the severity of disease and assess treatment outcomes.

There are several approaches to treat patients with ear deformity including surgical^3,4^ and nonsurgical intervention^5,6^. Otoplasty is often performed during childhood through adulthood or when the ear has reached its full size. Nonsurgical methods, such as splinting or rigid ear molding, take advantage of the plasticity of auricular cartilage during the neonatal period, correcting the deformity over the first month of life^5^. Early identification of ear deformity is crucial for the success of non-invasive ear molding^7^. At birth, high levels of circulating maternal estrogens result in increased levels of hyaluronic acid in ear cartilage, which increases the ear’s malleability and plasticity. These levels are highest in the 72 hours after birth and drop rapidly over the first 6 weeks of life^8^. As the estrogen level drops, the auricular cartilage stiffens, which prevents neonatal ear molding in achieving a long-term improvement. An alternative approach would require surgical correction^1,2^. To date, there are no reliable objective methods^1,2,9^ to evaluate ear shape anomalies and evaluation relies on subjective assessment. Therefore, an objective measure to assess ear deformity during the first month of life would be useful for practitioners and parents.

There are several types of congenital ear deformity, including Stahl’s ear, cup ear, and cryptotia, that can affect one or several structures of the ear. Developing mathematical modeling to identify ear deformity can be difficult due to the ear’s complex shape and composition. One solution is machine learning, which automatically recognizes patterns in the training data and builds models to predict future outputs. It is an innovative technique that has been shown to augment human intuition in data analysis. In particular, machine learning has been applied to several divisions of medicine including radiology^10–13^, ophthalmology^14,15^, dermatology^16,17^, and plastic surgery^18,19^ to detect patterns in data and assist in predicting disease or treatment outcome.

Deep learning, a branch of machine learning, has been equipped with feasible computational algorithms and its application has been successful in ear recognition^20,21^. The success comes from its deep layer structure. Among deep learning methods, convolutional neural networks (CNNs) excel in image analysis^22^ as they can capture local information within an image while reducing the complexity of the model. Therefore, CNNs have been successful in medical image analysis to automatically classify disease^16,23,24^ and segment anatomical regions^25,26^.

Training CNNs can be performed from scratch or by transfer learning. Training from scratch requires a large set of labeled training data, on the order of 1000 images per class, which is often lacking in the healthcare domain. In addition, training data this large is expensive and cumbersome to build^27^. In subspecialized medicine, such as plastic surgery, there is a lack of large number of standardized and annotated datasets due to the low number of patients seen at these clinics. Transfer learning allows for training with a source task if the target task is similar^27,28^, shrinking the necessary labeled training data. GoogLeNet has been pre-trained with 1.2 million images with more than 1000 object categories, and, therefore, has learned extensive features for a wide range of images.

In this study, we optimized a pre-trained CNN model, GoogLeNet^29^, to classify ears from 2D photographs as normal or abnormal. This study aims to evaluate the performance of CNN in classifying ear abnormality when compared to ground truth clinical diagnosis.

## Results

**Figure 1:**
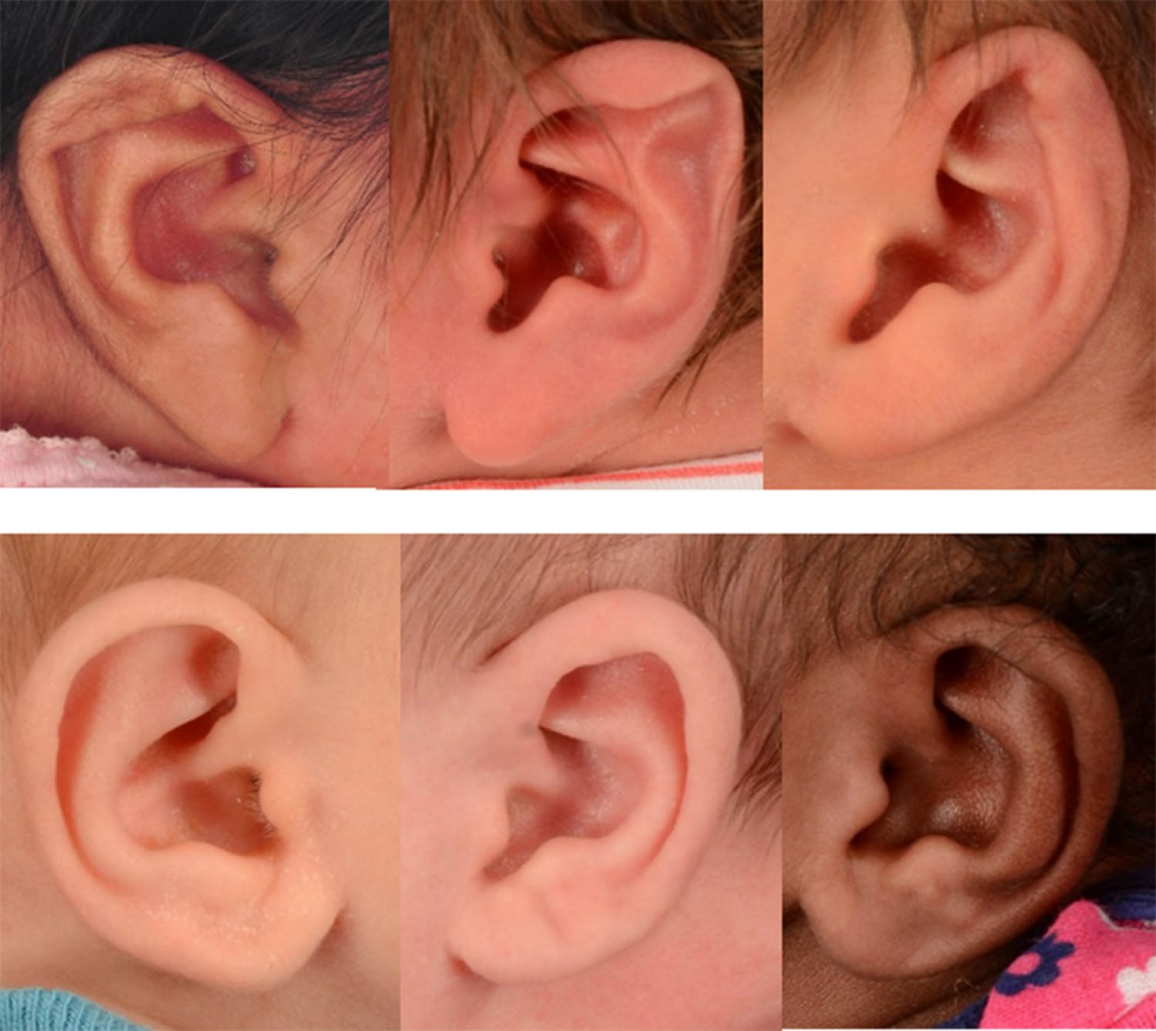
Sample photographs used to train the CNN model. A total of 274 photographs of abnormal ears (top panel) and 128 photographs of normal ears (bottom panel) were used.

**Figure 2:**
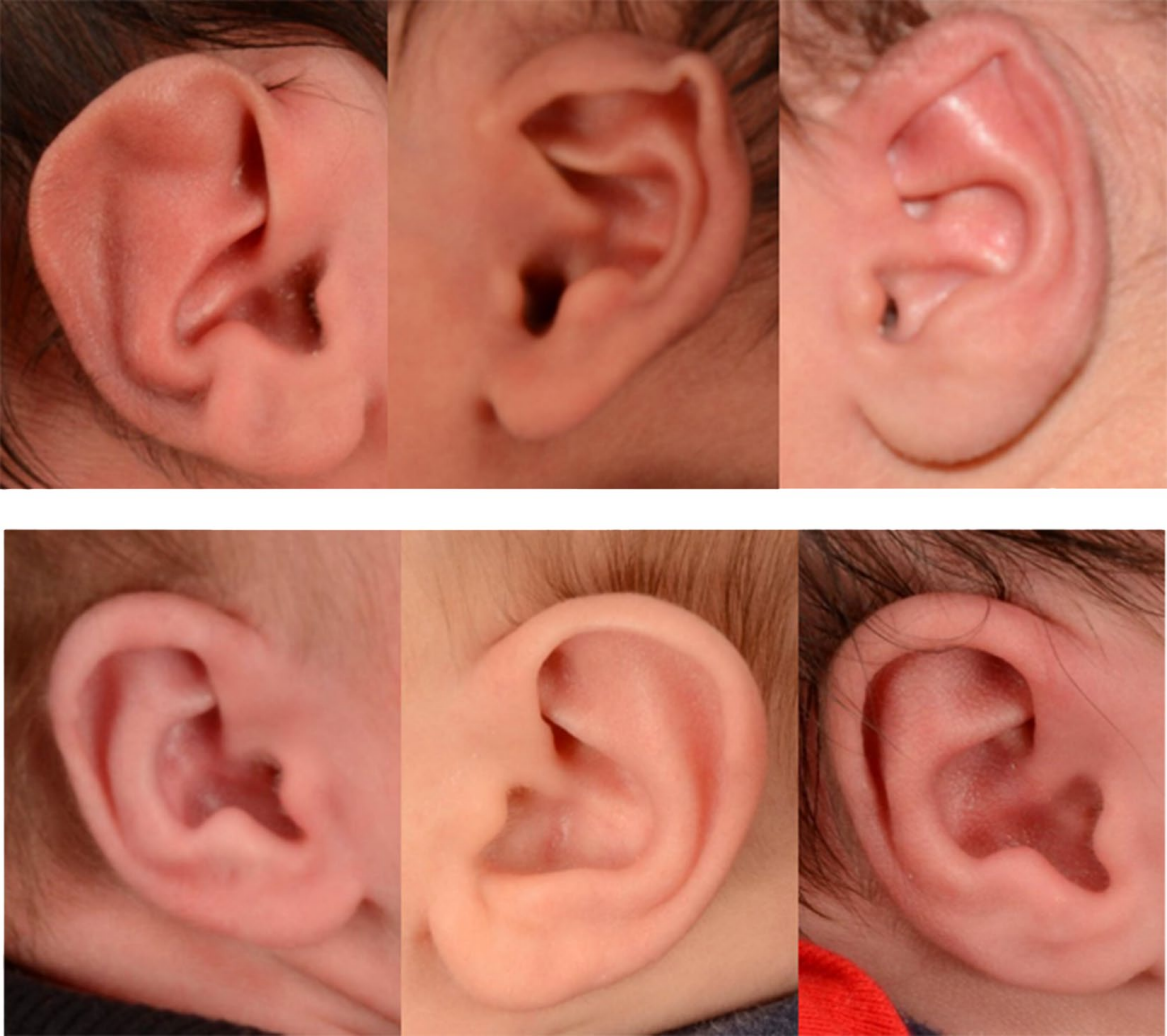
Sample photographs used to validate the CNN model. A total of 92 photographs of abnormal ears (top panel) and 43 photographs of normal ears (bottom panel) were used.

GoogLeNet was trained with 60% of the 671 photographs (274 abnormal vs 128 normal ears). Sample photographs of the training data can be seen in Fig. 1. It took about 2 hours to train GoogLeNet CNNs. After the training was completed, we tested our CNN model using the remaining 92 abnormal and 43 normal ears (Fig. 2).

Overall, our deep CNN model achieved 94.1% accuracy. In addition, the model achieved high precision 93.8%, sensitivity 97.8% and specificity 86.0%.

**Figure 3:**
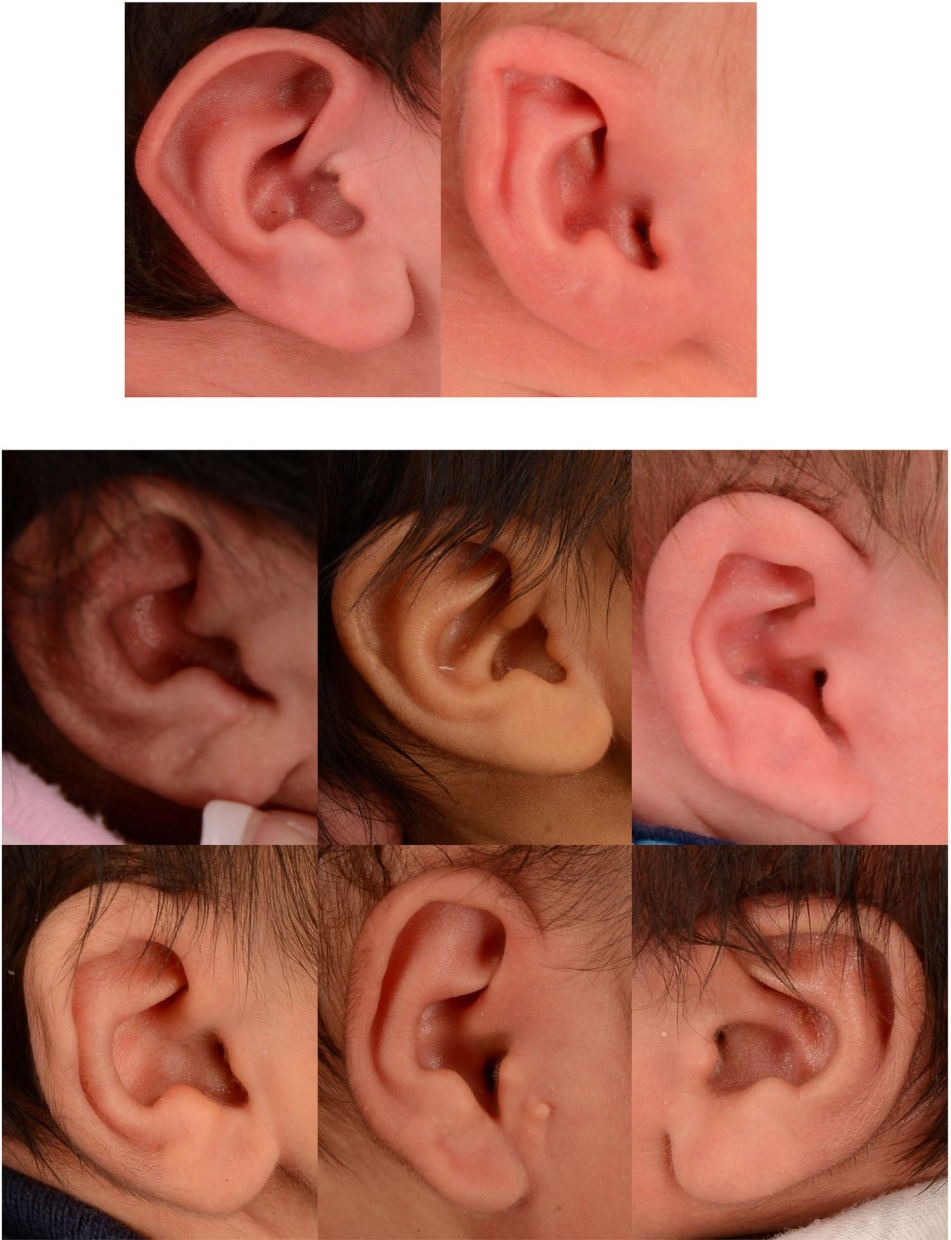
Misclassified photographs. The CNN model misdiagnosed 2 abnormal ears as normal (top row) and 6 normal ears as abnormal (bottom rows). The CNN model achieved 94.1% accuracy.

Of the 92 abnormal ears, the model classified 90 photographs correctly but misclassified 2 photographs. In addition, the model correctly classified 37 out the 43 normal ears. The misclassified photographs can be seen in Fig. 3.

## Discussion

In this study, we modified the GoogLeNet model to identify ear abnormalities from 2D photographs obtained for clinical practices. While these photographs are taken according to standard clinical photography^30^, they exhibit variability in factors such as the angle and zoom. Our CNN model identified ear abnormality with a high accuracy of 94.1%.

To our knowledge, this is the first study to use CNN to identify congenital ear abnormality. However, several mathematical models (edge detection, shape model and iterative closest point) have been developed for healthy ear detection or ear biometric recognition in adults^20,31^. While some models require user interaction for ear detections^32,33^, others developed automatic ear identification using image ray transform^34^. In addition, 3D images have been used for ear detection based on contour matching (achieved a 90.9% detection rate)^35^ or histograms of categorized shapes (achieved a 100% detection rate)^36^.

CNN has also been used for ear recognition from 2D photographs^20,37^. CNN achieved better accuracy (84.8%) when compared to traditional computer vision systems that uses feature extraction algorithm such as principal component analysis (accuracy 76.8%) and speeded up robust features (accuracy 76.8%)^20^.

The ability of artificial neural networks (ANN) to identify disease has been compared to classic methods in neurosurgery, oncology, and plastic surgery. A study using CNN to classify skin cancer achieved similar accuracy to board-certified dermatologists^16^. In 2018, the FDA approved the first medical device using deep learning to detect diabetic retinopathy. In neurosurgery, ANN accurately predicted endoscopic 3^rd^ ventriculostomy success^38^, patient satisfaction after laminectomy for lumbar spinal stenosis^39^, and in-hospital mortality in patients with traumatic brain injury^40^. In plastic surgery research, ANN predicted (accuracy 96%) whether a burn would heal within 14 days^41^.

This study shows that deep learning, such as a CNN, can provide objective assessment of ear deformity during the first month of life. A shared algorithm, if universally deemed accurate in identifying pathology and assessing treatment outcome, would be invaluable in making a diagnosis. Furthermore, computer programs or mobile applications equipped with deep learning models can potentially benefit early detection of disease (during the first month of life) outside of the clinic. For example, a parent or medical provider of a neonate who was unsure of whether there was an abnormality could submit a photograph for automated diagnosis.

This study’s neural network placed ears in the category of “normal” or “abnormal” as we do not have large annotated data available at our center to classify more specific pathology with respect to location on the auricle (e.g. lobule deformity, prominent ears, ear clefts, cup ear deformities, etc.)^42^. A future study might require a multicenter collaboration to obtain a sufficiently large series of data to train and test a neural network.

## Conclusion

The machine learning algorithm we used can extract features from pictures of ears and diagnose normal vs. abnormal with high accuracy. With further research, this could be a standardized tool to objectively evaluate ear intervention outcomes.

## Materials and Methods

This study was approved by the Institutional Review Board (IRB) at UT Southwestern Medical Center, and it was carried out in accordance with IRB guidelines and regulations. The IRB approved a waiver of informed consent given that our study is a retrospective review. After obtaining Institutional Review Board (IRB) approval, we retrieved 2D photographs of normal and abnormal ears that were taken as standard of care in our plastic and reconstructive surgery photography studio between 2009 and 2017. The photographs were retrieved using our database search engine^43^ to train and test the deep learning networks. Ears were labeled as “normal” or “abnormal” for the training and validation sets, based on the practitioner’s documentation of an ear molding intervention from the cohort of children who visit the Fogelson Plastic and Craniofacial surgery clinic for ear molding evaluation. Lateral view photographs were taken of one or both ears using a Nikon D90 with a Nikkor 24–85 mm f/3.5–4.5 lens. A PocketWizard transmitter and receiver were used to trigger the strobes.

A total of 671 ears (left or right side) were used, classified as follows: 457 photographs of patients with ear deformity and 214 photographs of patients with normal ears. Photographs were cropped to the ear boundary at a 4:5 aspect ratio and randomly divided into training (60%), validation (20%), and testing (20%) datasets. The model uses the pre-trained GoogLeNet architecture in Matlab. We modified the softmax classifier in the last layer of GoogleNet to perform binary classification (normal and abnormal). During the training, each training image was randomly scaled and translated per epoch to overcome an overfitting problem often caused by small training datasets. We optimized the batch size, number of epochs, and learning rate hyperparameters using the validation images. After testing several combinations of hyperparameters, we selected hyperparameters of batch size = 50; number of epochs = 300; and learning rate = 1e-4. Machine learning analyses were performed using an EVGA GeForce GTX 1080 with 8 GB onboard memory. The sensitivity, specificity, accuracy and precision were calculated.

### Institutional review board statement

IRB has approved this study.

## Data Availability

Data from this study are available to interested readers upon reasonable request.
